# Application of signal separation to diffraction image compression and serial crystallography

**DOI:** 10.1107/S1600576724011038

**Published:** 2025-02-01

**Authors:** Jérôme Kieffer, Julien Orlans, Nicolas Coquelle, Samuel Debionne, Shibom Basu, Alejandro Homs, Gianluca Santoni, Daniele De Sanctis

**Affiliations:** a European Synchrotron Radiation Facility, 71 avenue des Martyrs, CS 40220, 38043 Grenoble Cedex 9, France; b EMBL Grenoble, 71 avenue des Martyrs, CS 90181, 38042 Grenoble Cedex 9, France; Uppsala University, Sweden; The European Extreme Light Infrastucture, Czechia

**Keywords:** background extraction, serial crystallography, peak picking, lossy compression, diffraction data processing, signal separation

## Abstract

We present here a methodology for real-time analysis of diffraction images acquired at a high frame rate (925 Hz) and its application to macromolecular serial crystallography at ESRF. We introduce a new signal-separation algorithm, able to distinguish the amorphous (or powder diffraction) component from the diffraction signal originating from single crystals. It relies on the ability to work efficiently in azimuthal space and is implemented in *pyFAI*, the fast azimuthal integration library. Two applications are built upon this separation algorithm: a lossy compression algorithm and a peak-picking algorithm. The performances of both are assessed by comparing data quality after reduction with *XDS* and *CrystFEL*.

## Abstract 2

We introduce a new signal-separation algorithm for precise background assessment and describe its application to single-crystal image compression and serial crystallography data pre-processing.

## 1. Introduction

X-ray macromolecular crystallography is one of the most successful methods to determine the atomic structure of biological molecules. However, the achievable diffraction quality may often be limited by radiation damage. Although cryogenic conditions permit one to extend the lifetime of crystals in the X-ray beam and to increase the maximum absorbed dose before inducing damage, they may hinder analysis of physiologically relevant conformations. This limitation has renewed the interest in room-temperature macromolecular crystallography through applying a more drastic approach to overcome the radiation-damage problem by collecting data from thousands of small crystals, in what has become known as serial crystallography. First developed at X-ray free electron laser sources (Chapman *et al.*, 2011 ▸; Boutet *et al.*, 2012 ▸), the method is also currently applied at synchrotron sources (Diederichs & Wang, 2017 ▸; Gati *et al.*, 2014 ▸; Nogly *et al.*, 2015 ▸; Owen *et al.*, 2017 ▸).

### 1.1. Serial crystallography using synchrotron sources

Serial crystallography involves exposing thousands of small crystals to the X-ray beam only once in a serial way. Diffraction is collected with a very high flux density, in order to extract the most information from a single shot. This is in contrast with traditional rotational crystallography, where a complete dataset is collected from a single crystal rotated around one (or several) axis. These serial synchrotron crystallography (SSX) images represent a slice through the reciprocal space and thus intersect a lower fraction of the reciprocal space in comparison with rotational crystallography, as the crystal is still. To achieve a complete dataset, thousands of frames have to be collected, individually indexed and then merged. The high flux needed to collect all of the diffraction signal from a single crystal within a single exposure makes the SSX technique a good candidate to benefit from fourth-generation synchrotron sources, such as the new ESRF–EBS update (Chaize *et al.*, 2018 ▸). However, macromolecular crystallography beamlines are extremely specialized towards rotational data collection and thus require modifications to the experimental setup to perform SSX experiments. The synchrotron serial crystallography beamline ID29 at ESRF (Orlans *et al.*, 2025 ▸) has been built to have a dedicated environment to perform SSX experiments with a high flux (using a larger energy bandwidth with a multilayer monochromator), a high-speed chopper (to separate X-ray pulses), several sample-delivery methods and a fast detector.

### 1.2. Jungfrau 4M detector

Macromolecular crystallography has vastly progressed in recent decades with the introduction of photon-counting detectors (Broennimann *et al.*, 2006 ▸). With their absence of readout noise and their fast speed, the main limitation of photon-counting detectors is the achievable count rate, *i.e.* how fast the electronics of a pixel are able to count arriving photons.

The ID29 beamline features a Jungfrau 4M detector. Unlike photon-counting detectors such as the Eiger detector (Casanas *et al.*, 2016 ▸), the Jungfrau detector (Mozzanica *et al.*, 2016 ▸) is an integrating detector. Thus it is not limited by the count rate, even under the very intense flux expected when recording Bragg peaks. To cope with this photon density, every single pixel implements an automatic gain-switching mechanism (three levels), which offers a precision of the order of one-third of a kiloelectron volt in the higher-gain mode, a precision of the order of one photon at the intermediate level and the ability to cope with thousands of photons in the lower-gain mode. Moreover, the Jungfrau detector is able to operate at 2 kHz, which means the image must be read every half a millisecond. Since the Jungfrau detector is an integrating detector, dark-current and flat-field corrections have to be applied: every pixel has three ‘pedestals’ and three gain values (one for each gain level). This large number of parameters per pixel makes the pre-processing of the raw signal challenging at full speed: the signal from a single pixel, initially packed with 16 bits, gets expanded to 32 bits (floating point or integer value), doubling the required bandwidth and the storage size (Leonarski *et al.*, 2020 ▸). The Jungfrau 4M detector at the ID29 beamline operates at 1 kHz, with pace imposed by the chopper and synchronized with the photon bunches from ESRF. At nominal speed, the detector will produce 16 GB of pre-processed data per second, making the data analysis and storage extremely challenging.

### 1.3. Requirements for online data processing

Serial crystallography is one of the techniques where online data processing is likely to have the most impact: millions of images are collected and existing detectors already saturate the fastest storage systems, not even considering the cost of this storage. Beside this, only a small percentage of the frames are expected to contain diffraction signal and, out of them, a fraction will be indexed, integrated and thus useful to solve the protein structure. Efficient processing of raw images is therefore essential for SSX.

This article describes the methodology that has been developed for real-time analysis of diffraction images acquired with the Jungfrau detector at the ID29 beamline of ESRF. The algorithms described here were developed in OpenCL (Khronos, 2008 ▸) using the *pyFAI* software package (Kieffer & Wright, 2013 ▸) and are currently in use at ESRF and at other facilities.

The data processing stategy involves five levels of increasing complexity: (1) image reconstruction with pedestal correction, (2) a veto algorithm to sieve out images with poor signal, (3) saving of only pixels with diffraction signal, (4) precise location of peak position with indexing (Gasparotto *et al.*, 2024 ▸) and (5) real-time integration of diffraction peaks.

Reconstruction and pedestal correction have already been described by Debionne *et al.* (2022*a* ▸). This article will focus on the subsequent steps – the detection of signal is addressed in Section 2, sparse data compression in Section 3 and peak finding in Section 4 – before drawing some conclusions in Section 5.

## 2. Algorithm for the separation of the amorphous background from the Bragg peaks

### 2.1. Background scattering

The simplest implementation of Bragg peak separation is to assume that the background signal originates from scattering of amorphous material giving an isotropic signal that ideally presents only smooth variations. Before background subtraction, the raw signal has to be corrected for dark noise and for any systematic anisotropic effects such as polarization corrections. Unlike X-ray free electron lasers, a synchrotron X-ray beam is better characterized in energy and shows little to no pulse-to-pulse variability. All anisotropic correction can be easily modeled and taken into account. The same method can be extended to separate Bragg peaks from powder diffraction if the powder signal is isotropic, *i.e.* without preferred orientation.

The initial implementation of signal separation in *pyFAI* (Kieffer & Wright, 2013 ▸) relies on a 2D polar transform followed by a median filter in the azimuthal dimension to calculate the amorphous scattering curve. Although this method has been successfully used for large dataset analysis (Bordet *et al.*, 2021 ▸), it presents four major drawbacks: (i) the 2D averaging mixes the signal originating from several pixels and blurs the signal; (ii) pixel splitting is needed to leverage the Moiré effect in the 2D averaging, but this further increases the blurring (Fang *et al.*, 2020 ▸); (iii) the 1D curve obtained after the application of the median filter shows sharp jumps from one azimuthal bin to its neighbor; and (iv) the median filter is computationally heavy since it is required to sort out every azimuthal bin.

We have improved on this by developing a new efficient way of performing the azimuthal averaging (including the associated uncertainty propagation).

### 2.2. Efficient azimuthal averaging and uncertainty evaluation

#### 2.2.1. Pre-processing

The first step of the analysis involves applying a pixel-wise correction for dark current and several normalization corrections (Kieffer *et al.*, 2020 ▸): 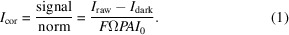
In equation (1), the numerator (referred to as ‘signal’ hereafter) is given by the subtraction of the dark current *I*_dark_ from the detector’s raw signal *I*_raw_. The denominator (hereafter ‘norm’) is a normalization factor composed of the product of *F*, a factor accounting for the flat-field correction; Ω, the solid angle subtended by a given pixel; *P*, the polarization-correction term; and *A*, the detector’s apparent efficiency which is related to the incidence angle of the photon on the detector plane. For integrating detectors, photons with high energy see a longer sensor path with larger incidence angles compared with the normal thickness, and thus they have a higher detection probability. The intensity is also normalized by the incoming flux *I*_0_ but, it being independent of the pixel position, this correction can be applied when convenient.

#### 2.2.2. Azimuthal averaging

Historically, azimuthal averaging has been implemented using histograms (Hammersley *et al.*, 1996 ▸). Since the geometry of the experimental setup is fixed during the acquisition, a look-up table listing all pixels contributing to each azimuthal bin can be built and used to speed up calculations (Kieffer & Ashiotis, 2014 ▸). The azimuthal transformation being a linear transformation, it can be implemented as a matrix multiplication, with a sparse matrix representing the transformation and a dense vector containing the flattened view of the diffraction image. The compressed sparse row matrix representation is preferred for its efficiency in performing dot products with dense vectors (Toledo, 1997 ▸). The coefficients *c*_*i*,*r*_ of the matrix are the fraction of area of a pixel *i* falling into the radial bin *r*. In the case where pixel splitting is deactivated, these coefficients (*c*_*i*,*r*_) are always one (and zero elsewhere) since each pixel contributes to a single bin. Sparse-matrix multiplication can be used to efficiently sum values for all pixels belonging to the same bin. The summed signal divided by the summed normalization provides the weight-averaged intensity over all pixels falling in the bin at the distance *r* from the center, as formalized in equation (2): 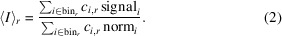


#### 2.2.3. Uncertainty evaluation from Poisson distribution

Photon-counting detectors, such as Eiger detectors, suffer from hardly any error beside the counting uncertainty, which is often referred to as Poisson statistics. This statistical law is described by a single parameter λ, which is related to the mean μ and standard deviation σ from a normal distribution by λ = μ = σ^2^. Other sources of noise, like the dark-current noise in the case of an integrating detector, superimpose quadratically on the Poisson noise, as presented in equation (3): 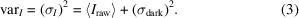
During the azimuthal integration, the coefficient of the sparse matrix needs to be squared in the numerator when propagating the variance [equation (4)] to have uncertainties σ proportional to the fraction of the pixel considered: 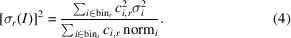
One should distinguish the uncertainty of the mean (sometimes referred to as the standard error of the mean, sem), which describes the precision with which the mean is known [and is described by Kieffer *et al.* (2020) ▸], from the uncertainty of the pixel value (often referred to as standard deviation, std), which describes the uncertainty with which the pixel value is known. These two values differ only by the square root of the number of measurements in the case of an arithmetic mean: sem = std/(*N*)^1/2^, with *N* being the number of pixels contributing to the bin. When considering the weighted average, the previous formula becomes
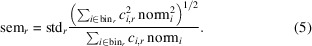
Thus, the more data points collected, the more precisely the mean value is known, but the uncertainty for a given point remains the same. Since this article focuses on the uncertainties of pixel values, the standard deviation will systematically be used from here on.

#### 2.2.4. Uncertainty evaluation from the variance in a bin

Unlike photon-counting detectors, most detectors do not follow the Poisson distribution, and therefore the definition of a relation σ^2^ = *f*(*I*) is not simple, if possible at all. The integrating Jungfrau detector has a complex gain-switching mechanism (Leonarski *et al.*, 2020 ▸), which makes this equation complicated. Therefore, a generic approach is proposed to measure the variance in every single azimuthal bin.

When considering the diffraction of an isotropic compound (liquid, amorphous or perfect powder), all pixels contributing to the same radial bin should see the same flux of photons (after correction of anisotropy like polarization), and the deviation of their intensities can be used to estimate the uncertainty. This approach is of course limited when considering the signal coming from a few large crystallites (where rings become spotty) but it provides an upper bound for the uncertainty. Variances (thus standard deviations) are usually obtained in a two-step procedure: one pass to calculate the average value [equation (2)] and a second to calculate the deviation from the average [equation (6)]. This double-pass approach can be implemented using sparse-matrix multiplication. This requires twice the access to each pixel value, and extra storage space, but it is numerically robust (*i.e.* not prone to numerical-error accumulation). 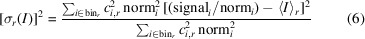
and 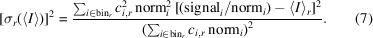
Single-pass implementations of variance calculation are faster than double-pass ones since they access pixels only once and offer, in addition, the ability to perform parallel reductions (Blelloch, 1996 ▸), *i.e.* work with blocks of pixels. Schubert & Gertz (2018 ▸) present a complete review on the topic, which introduces a formalism adapted here for crystallography. First, assume that the weight of a pixel is ω_*i*_ = *c*_*i*_ norm_*i*_. If *P* is a partition of the ensemble of pixels falling into a given azimuthal bin, let Ω_*P*_, *V*_*P*_ and *VV*_*P*_ be the sum of weights [equation (8)], the weighted sum of *V* [equation (10)] and the weighted sum of deviation squared [equation (11)] over the partition *P*, respectively: 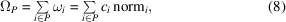

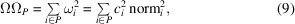

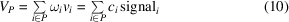
and 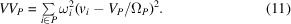
The weighted average and associated variances are then expressed as 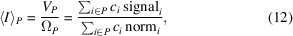

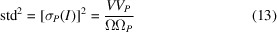
and 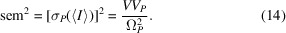
In this formalism, equations (2) and (12) on one side and equations (6) and (13) on the other are actually equivalent. Schubert & Gertz (2018 ▸) present a way to perform the union of two sub-partitions *A* and *B* of a larger ensemble that opens the doors to parallel reductions, which are especially efficient when implemented on a GPU: 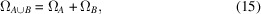

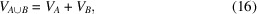

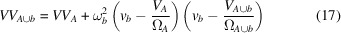
and 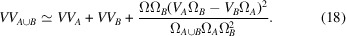
While equations (15) and (16) are trivial, equation (17) describes the nominator of the variance of an ensemble when adding an extra member *b* to *A*. Unfortunately, the slight difference of formalism between Schubert & Gertz (2018 ▸) and this work prevents some simplification from occurring, and leads to the approximate numerator of the variance (*VV*) in the case of the union of two ensembles *A* and *B* [equation (18)], used in OpenCL reduction.^1^ However, a numerical stability issue can arise from it when *V*_*A*_ or *V*_*B*_ are very small, and this issue is addressed by using double-precision arithmetic when implemented on a CPU and double-word arithmetic when running on a GPU (Joldes *et al.*, 2017 ▸).

#### 2.2.5. Comparison of uncertainty models

**Figure 1:**
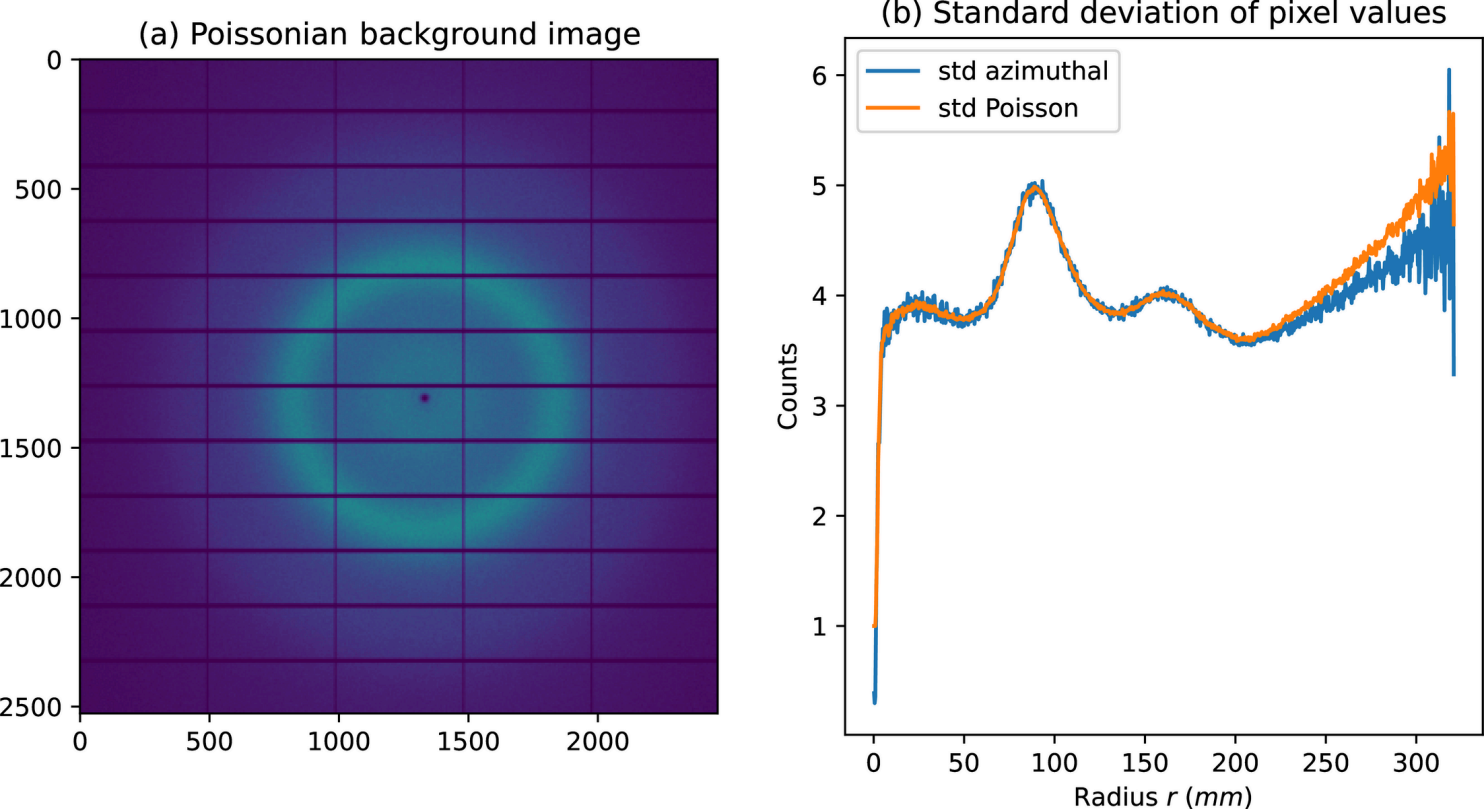
(*a*) A simulated diffraction frame of a Pilatus 6M detector with pure azimuthal Poisson noise and (*b*) uncertainties for pixel intensity as measured with the distance to the mean (azimuthal model, blue) or from the Poisson model (orange).

Fig. 1 ▸(*b*) presents the uncertainties (for the pixel value) as calculated from a background frame with pure Poisson noise [Fig. 1 ▸(*a*), synthetic data] using the two algorithms previously described: the Poisson model or calculated from the variance in the azimuthal bin. While the two curves show similar amplitude, except in the corner of the detector where very few pixels contribute to each of the azimuthal bins, the variability of the ‘azimuthal’ model is much greater from one bin to the neighboring one.

### 2.3. Histogram intensity

**Figure 2:**
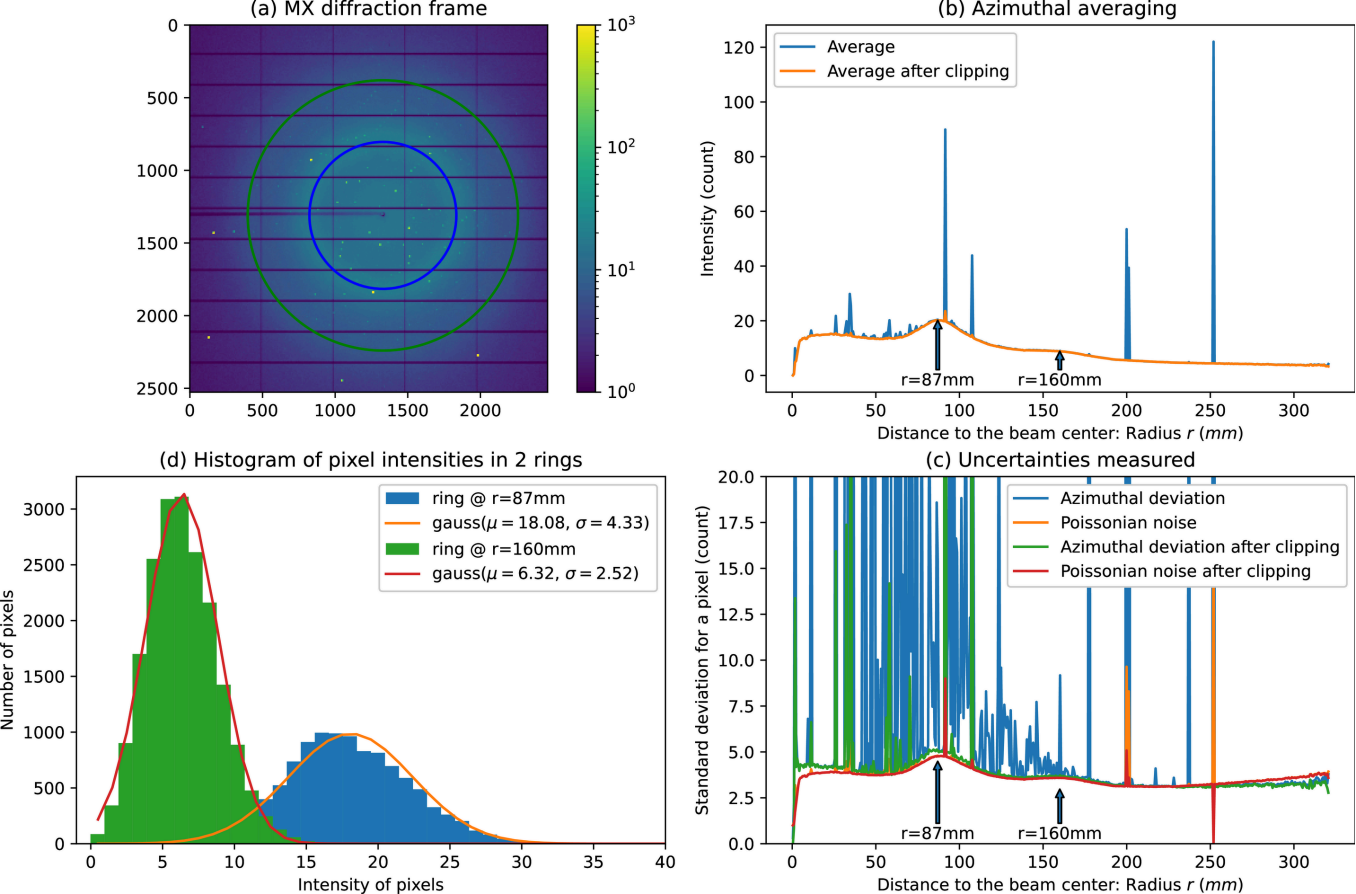
(*a*) A single-crystal diffraction frame obtained from insulin with a Pilatus 6M detector with (*b*) the azimuthally averaged signal before and after clipping data. (*c*) Uncertainties when calculated assuming a Poissonian error model (orange, red) or when measuring the deviation within all pixels in a ring (green, blue). (*d*) A histogram of intensities for two rings at *r* = 87 mm and *r* = 160 mm from the beam center with the distribution fitted as Gaussian curves: 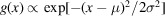.

Fig. 2 ▸ presents the diffraction from a single crystal of insulin collected with a Pilatus 6M detector [Fig. 2 ▸(*a*)] and several curves obtained from azimuthal integration of these data. Fig. 2 ▸(*b*) is the azimuthally integrated signal (blue curve), where Bragg peaks are seen as spikes on top of a smooth background. Fig. 2 ▸(*c*) presents the uncertainties measured according to the Poisson distribution (orange curve) or the deviation in the ring (blue curve). The latter presents much larger values since Bragg peaks contribute a lot to the deviation despite them representing few pixels: this highlights the sensitivity of the mean/std to outliers. Fig. 2 ▸(*d*) presents histograms of pixel intensity for pixels lying at 87 and 160 mm from the beam center. Each of these histograms is composed of a bell-shaped distribution with a few positive outliers, which are usually Bragg peaks. The histograms in Fig. 2 ▸(*d*) have been fitted with Gaussian curves, and the center (μ) and the width (σ) of the curves match roughly with the average [in Fig. 2 ▸(*b*)] and uncertainties [in Fig. 2 ▸(*c*)].

The core idea of the algorithm for background extraction is to model the distribution of background pixels. Unlike Bayesian statistics (Sivia & Skilling, 2006 ▸) where the cost function is usually tuned to give less weight to outliers, here these outliers are simply flagged and discarded. Positive outliers can reasonably be assigned to Bragg peaks and negative outliers to shadows or defective pixels. The distribution is recalculated after discarding pixels for which the intensity differs from the average value by more than *n* times the standard deviation, 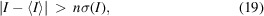
where *n* is called the signal-to-noise ratio (SNR). This clipping re-centers the distribution of the remaining pixels since the mean is sensitive to outlier values. The orange plot in Fig. 2 ▸(*b*) presents the average after having discarded these outliers, and the red and green curves of Fig. 2 ▸(*c*) are the uncertainties calculated after this clipping. After clipping, the average and uncertainty curves have lost most of their spikes, which means that most Bragg peaks and shadowed pixels have been discarded.

Of course this works only if the background signal is isotropic, ideally smoothly varying, and there are many more background pixels than peaks or shadowed pixels. While shadows can be handled with a user-defined mask, anisotropy in the background scattering, as is sometimes observed with certain stretched plastic films in fixed-target mode, is much more challenging and will not be addressable with this analysis.

### 2.4. Sigma clipping

The sigma-clipping algorithm consists of applying this outlier rejection several times. If the initial distribution is mono-modal, this algorithm gradually forces the data to be sampled symmetrically around the maximum probability, which is likely to look like a normal distribution. If the initial distribution is more complicated (typically multi-modal), the larger standard deviation will prevent most outlier pixels from being rejected, making it more conservative. The sigma-clipping algorithm uses two parameters: the number of iterations and the rejection cut-off (SNR). Despite the execution time being proportional to the number of iterations of sigma clipping, iterations should continue until no more outliers are found, so that the background data can be treated assuming a normal distribution. Since the loop exits as soon as no more outliers are discarded at the clipping step, having an arbitrary large number of iterations is not really an issue for the execution time and the number of actual iterations is usually few (3 are commonly observed).

#### 2.4.1. Limits of the Poissonian approach

The evaluation of uncertainties based on the variance within a radial shell (azimuthal model) was developed after numerical artefacts were discovered while performing sigma clipping with a Poissonian approach. Some azimuthal bins showed no pixel contribution at all and thus appeared without any mean or uncertainties, jeopardizing the complete background-extraction algorithm. This artefact was directly linked to the usage of Poisson statistics and can be demonstrated with a simple distribution of two pixels with values 1 and 199. The mean of this distribution is 100 and the standard deviation is also close to 100, while the uncertainty derived from a Poissonian law would be close to 10 (*i.e.* 100^1/2^). With the azimuthal error model, both pixels are 1σ from the mean, while with the Poissonian error model, pixels are at 10σ. This explains why bins featuring strong Bragg peaks on top of a low background got completely emptied of any contributing pixels when sigma clipping was performed assuming Poissonian noise.

Unlike the Poisson error model, the azimuthal model provides uncertainties that are resilient to diffraction data coming from several types of samples but show much more variability from one bin to its neighbor [Fig. 1 ▸(*b*)]. The package *pyFAI* introduces a hybrid error model that uses the azimuthal error model for the sigma-clipping stage, which trims the ensemble of pixels to become mono-modal. The uncertainties are then calculated using the Poisson error model on the trimmed ensemble.

#### 2.4.2. Clipping threshold

The clipping threshold can be automatically calculated via a variation of Chauvenet’s criterion (Maples *et al.*, 2018 ▸) where one would accept to discard only a single pixel in a ring with a signal already following a normal law. Thus, the threshold value is adapted to the size of the distribution, *i.e.* the number of pixels in each ring [equation (20)], which can reach several thousands and shrinks with iteration: 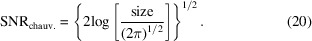
Typically, the numerical value for this cut-off varies from 2 to 4.

The worse-case scenario for sigma clipping corresponds to an initial distribution that is very far from a normal distribution, like the bimodal distribution seen in the previous section. Another challenging situation occurs close to the detector corners where the background signal is low and the size of the distribution is decreasing. For example, this cut-off parameter increases from 2.7 to 3.5 when the size of the ensemble increases from 100 to 1000 elements. So, for a Poissonian detector and a low count rate of 1 (λ = μ = σ^2^ = 1), any pixel with intensity greater than 4 is discarded with an ensemble of 100 pixels, while the cut-off is greater than 5 for an ensemble of 1000 pixels.

## 3. Application to lossy image compression for X-ray diffraction

Diffraction images from protein crystals usually exhibit an isotropic background on top of which Bragg peaks appear (discarding any diffuse scattering). The sigma-clipping algorithm can be used to select the background level and, more importantly, the associated uncertainty. This lossy compression algorithm consists of saving only pixels where intensity is above the average background value (μ) plus *n* standard deviation (σ).

**Figure 3:**
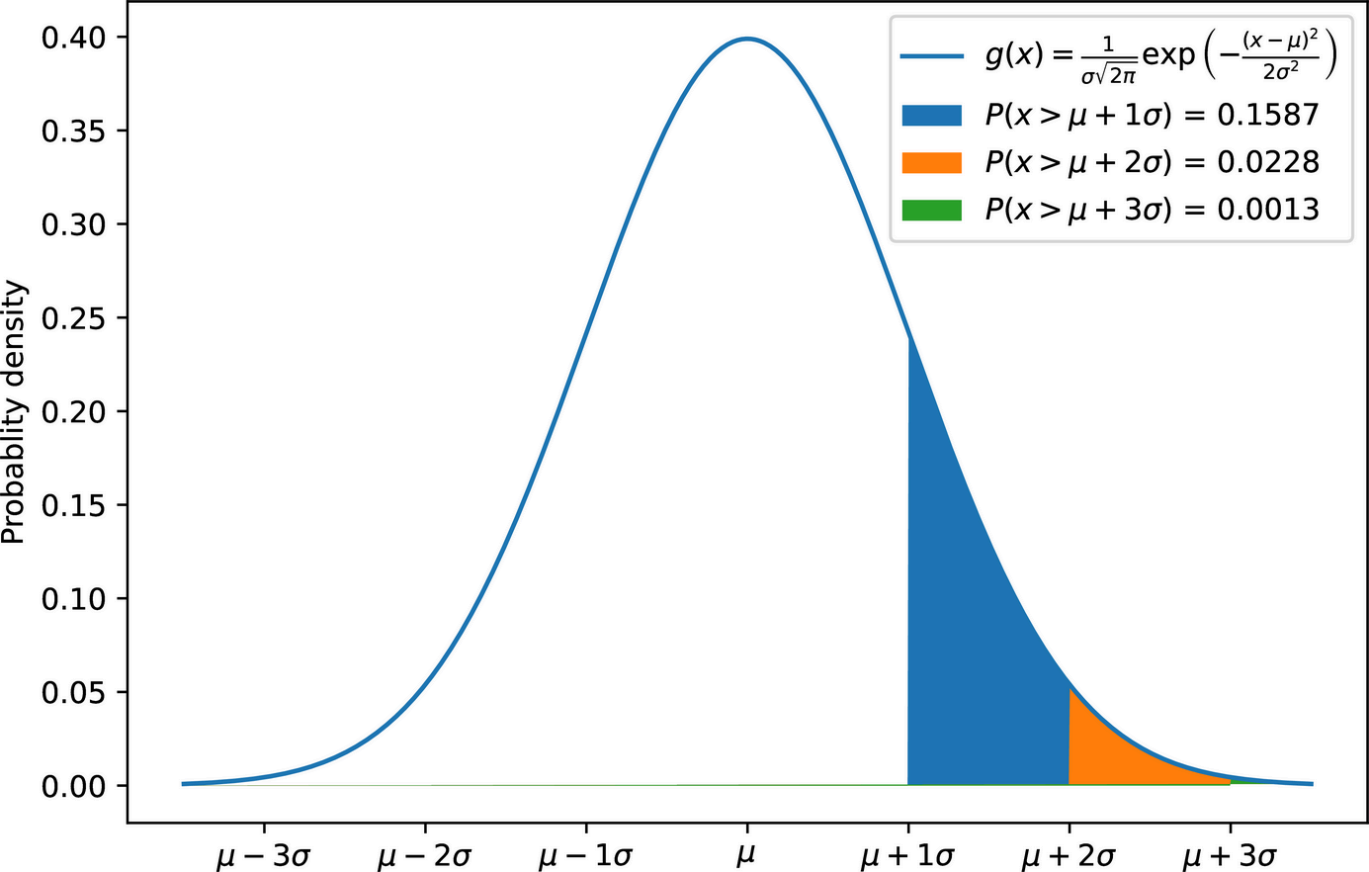
Normal distribution and probability of having pixels with intensities above a certain threshold. The cut-off parameters govern how much signal is integrally kept, thus the achievable compression rate on the one hand and the limit of quality of data on the other. It is the user’s responsibility to set this threshold wisely.

The decompression simply restores these intense pixels and builds a smooth background for the missing ones (possibly with noise). The cut-off value *n* (also called SNR_pick_) controls the amount of data to store. It is linked to the compression ratio. Assuming a normal distribution has been enforced at the sigma-clipping stage, 16% of the pixel is recorded with *n* = 1, 2.3% for *n* = 2 and only 0.13% for *n* = 3, as depicted in Fig. 3 ▸.

The compressed data format consists of (1) pixel intensities and positions for pixels worth saving, (2) the average level of the background and associated uncertainties as a function of the distance to the beam center, and (3) the distance to the center of every pixel.

Since diffraction analysis software performs some kind of noise-level analysis, the background signal has to be regenerated with intensity and noise similar to the original data.

### 3.1. Sparsification/compression

The sigma-clipping algorithm was originally written for real-time sparsification of single-crystal diffraction data, and its integration into the Library for Image Acquisition Version 2 (Lima2) detector control system (Petitdemange *et al.*, 2018 ▸) for the Jungfrau 4M detector used at ESRF ID29 is described by Debionne *et al.* (2022*b* ▸). The constraints of real-time analysis meant that we had to develop code running on GPUs, since these devices are several times faster than equivalently priced processors. All algorithms were developed in OpenCL (Khronos, 2008 ▸) and implemented in the *pyFAI* software package (MIT license, available on github). A command-line tool called *sparsify-Bragg* has been available for testing off-line since version 2023.01.

All the pixel coordinates and intensities are stored in an HDF5 container (The HDF Group, 2000–2021 ▸) following the NeXus convention (Könnecke *et al.*, 2015 ▸), together with a snippet of Python code explaining how to rebuild the dataset. All sparse datasets (averaged and uncertainties curves, pixel coordinates, *etc*.) are compressed with *bitshuffle-LZ4* (Masui *et al.*, 2015 ▸) lossless compression.

### 3.2. Densification/decompression

Since no crystallographic software package can deal with this sparse format (yet), a densification code was developed to regenerate initial frames and the *densify-Bragg* program was made available as part of the *FabIO* (Knudsen *et al.*, 2013 ▸) software package (MIT license). The source code is deposited on github (https://github.com/silx-kit/fabio) and *densify-Bragg* has been available since version 2022.12 via usual channels like pip or conda install. The software constraints for this densification code are very different from those for sparsification since this code can be used by users after they have left the beamline. For this reason, *densify-Bragg* was optimized to run on a multi-core CPU. Maybe an important consideration is whether, regardless of the file format, it is necessary to reconstruct the background or not. In fact, some crystallographic reduction programs like *CrysAlisPro* (Rigaku Oxford Diffraction, 2015 ▸) provide a better result with noise-less background while *XDS* (Kabsch, 2010 ▸), which performs a deep noise analysis, needs to have the noisy background properly restored. Shaded regions are never reconstructed properly and should be masked adequately in the reduction software.

Future development will focus on HDF5-plugins able to provide access to densified images from their sparse representation using a user-defined function in HDF5 (Real & de Bayser, 2021 ▸). This will allow any analysis software (already able to read HDF5 files) to treat sparse data as if they were dense, removing the burden of densifing the images from the users.

### 3.3. Performance on a single-crystal protein dataset

The performance for a lossy compression algorithm was evaluated along many directions: compression and decompression speeds, compression ratio, and degradation of the recorded signal. In the following example, we present the sparsification of an egg-white lysozyme (HEWL) dataset obtained using traditional oscillation data collection. Data were collected on an Eiger 4M detector (Dectris, 2014 ▸), selected for its similarity in size and performance to the Jungfrau detector. These data were then densified again to regenerate the data and processed in *XDS* (Kabsch, 2010 ▸). Data-quality indicators were finally compared between the original dataset and the one that went through the lossy compression presented here.

#### 3.3.1. Compression ratio

After sparsification (picking cut-off: 2σ; error model: Poissonian), the size of the dataset is still 103 MB, which represents a 15× compression ratio compared with the standard procedure. For conformance with the state of the art, the reference dataset was re-compressed using the *bitshuffle-LZ4* algorithm (Masui *et al.*, 2015 ▸), for which the 1800 frames have a size of 1500 MB (instead of the 5000 GB of the original files compressed in LZ4).

The maximum theoretical compression ratio for 2σ is 22× (Fig. 3 ▸, neglecting the storage of the background data and effects of the lossless compression). To evaluate the effective maximal compression ratio, the dataset was median filtered along the image stack to produce an image without peaks. A background dataset of 1800 such images sparsifies into an 11 MB HDF5 file, which represents a compression ratio of 136×. Indeed, only 19 pixels were saved per frame and the compressed numerical values are mostly the same, which facilitates compression with *bitshuffle-LZ4*.

For ESRF-ID29, where the Jungfrau 4M detector can operate close to 1 kHz, the pedestal + gain pre-processing converts 16-bit integers into 32-bit floating point values, doubling the bandwidth for data saving. The detector outputs the data via 8× 10 Gbit s^−1^ network links and the storage is performed via a single 25 Gbit s^−1^ link, making a minimum compression ratio of 6.4×.

In production conditions, ID29 users have to trade between:

(*a*) Detector speed: operate at a lower speed (231 Hz) to save the pre-processed data.

(*b*) Energy resolution: floating data compress badly, so they are better stored as integers, where the number of analog-to-digital units (ADUs) per photon can be tuned. With 1 ADU per photon, one gets the best compression rates but loses the sub-photon energy resolution of the Jungfrau detector.

(*c*) Using the burst mode: acquire only shorter datasets and make pauses to let the data reach the disk in the central storage.

(*d*) Discarding pixels of lower intensity: this is a new possibility offered by this algorithm. A cut-off at 1.5σ should already provide the 6.4× compression ratio needed (Fig. 3 ▸).

#### 3.3.2. Compression speed

The compression speed has been measured on a computer designed for online data reduction of the Jungfrau detector (Debionne *et al.*, 2022*b* ▸): an IBM AC922 using two Power9 processors and two Nvidia Tesla V100 GPUs. The sequential execution of the code on the GPUs takes about 4 ms to process one image, and uses one single CPU core and a GPU. In production conditions, two such computers each drive two GPUs (for a total of 4 GPUs), allowing one to use the detector at its nominal speed close to 1 kHz. The main bottleneck remains the networked saving of the different files: pre-processed files, sparse files, peak positions, accumulated frames,…. Not all can be saved at all times when operating at full speed.

#### 3.3.3. Decompression speed

The decompression of these data should typically be performed on a standard workstation (here, running two Intel Xeon Gold 6134 CPUs @ 3.20 GHz): the reconstruction speed takes 30 s for the full dataset, while writing of the densified dataset (with *bitshuffle-LZ4* compression) takes 45 s. Densification is thus faster than writing on disk. The reading time of the input sparse dataset is negligible (<2 s).

#### 3.3.4. Quality of the restored dataset

The densified dataset was processed via *XDS* and the summary indicator for the quality of the results was compared with that from the reduction of the original dataset. Since these integrators are measured on integral peaks with *I*/σ > 3 and the sparsification was performed with a cut-off of 2, these results should be almost unaffected, which is confirmed in Table 1 ▸.

Of course, these data were collected on a test sample with a very intense signal; but the example demonstrates that the algorithm does not destroy the signal. However, with more challenging samples, exhibiting lower *I*/σ, the threshold for picking pixels has to be lower to ensure all pixels relevant for subsequent analysis are actually preserve: unless, and as described by Galchenkova *et al.* (2024 ▸), this sparsification would be detrimental for the quality of the reduced data.

### 3.4. Influence of the sparsification on the quality of serial crystallography data acquired with an Eiger detector

Tiny crystals of HEWL + gadolinium were deposited on a SiN membrane and this membrane was scanned on the ID30A3 beamline at ESRF (massif3) using an Eiger 4M detector. The dataset consists of 11 637 frames of images, out of which 11 512 were properly indexed.

#### 3.4.1. Quality-indicator degradation

**Figure 4:**
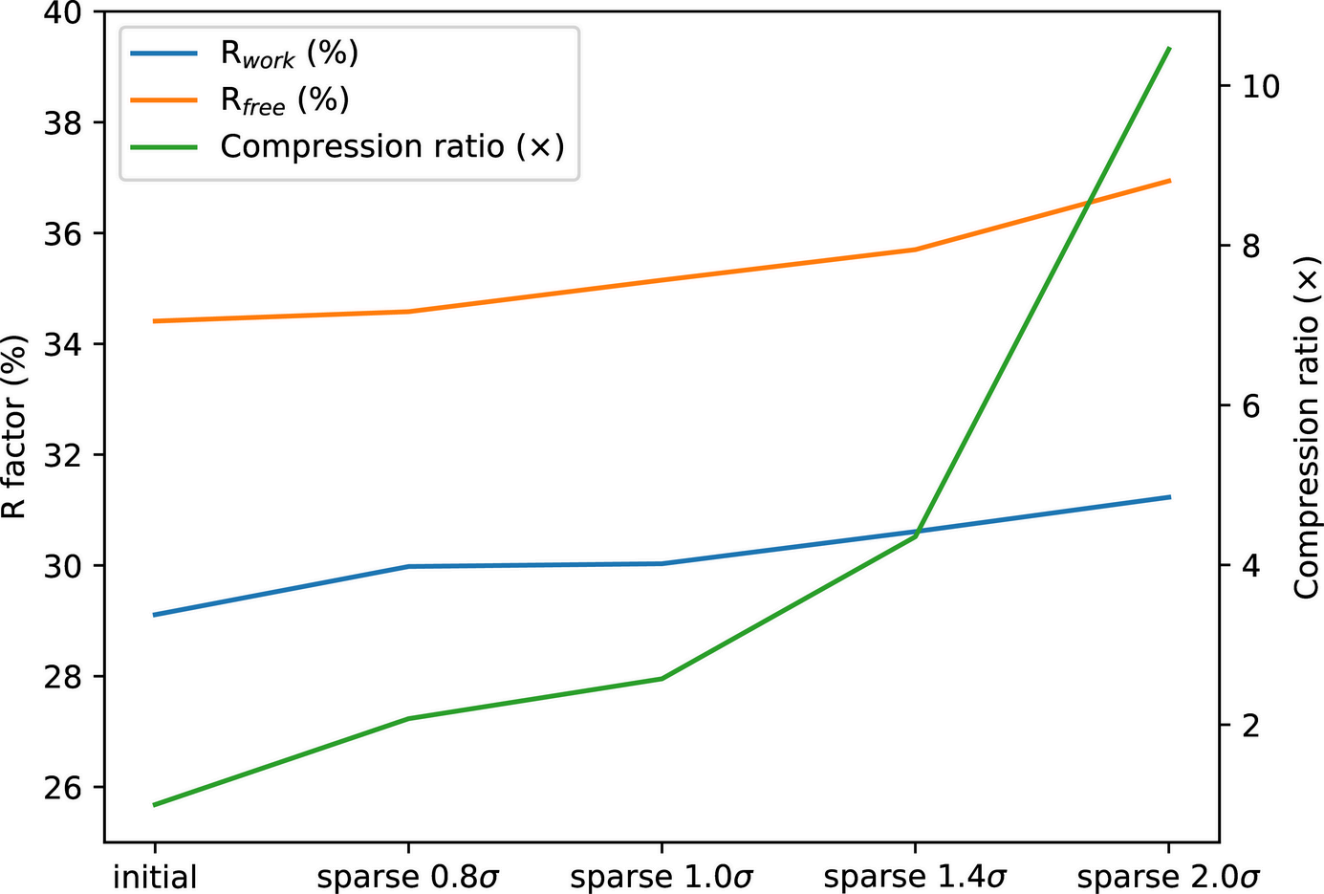
Degradation of *R*_work_ and *R*_free_ crystallographic quality indicators on the integral dataset of HEWL + Ga (11k frames) and actual compression rates, when increasing the levels of sparsification.

The *R*_free_/*R*_work_ quality indicators (Brünger, 1992 ▸) were calculated for the initial dataset and compared with the same dataset sparsified at 0.8σ, 1.0σ, 1.4σ and 2.0σ, and subsequently re-densified (Fig. 4 ▸).

As expected, the crystallographic quality indicators show a gradual degradation with increasing sparsification but *R*_free_ shows little degradation with little sparsification (0.8σ, compression ratio of 2×).

#### 3.4.2. Quality indicators as a function of the resolution shell

**Figure 5:**
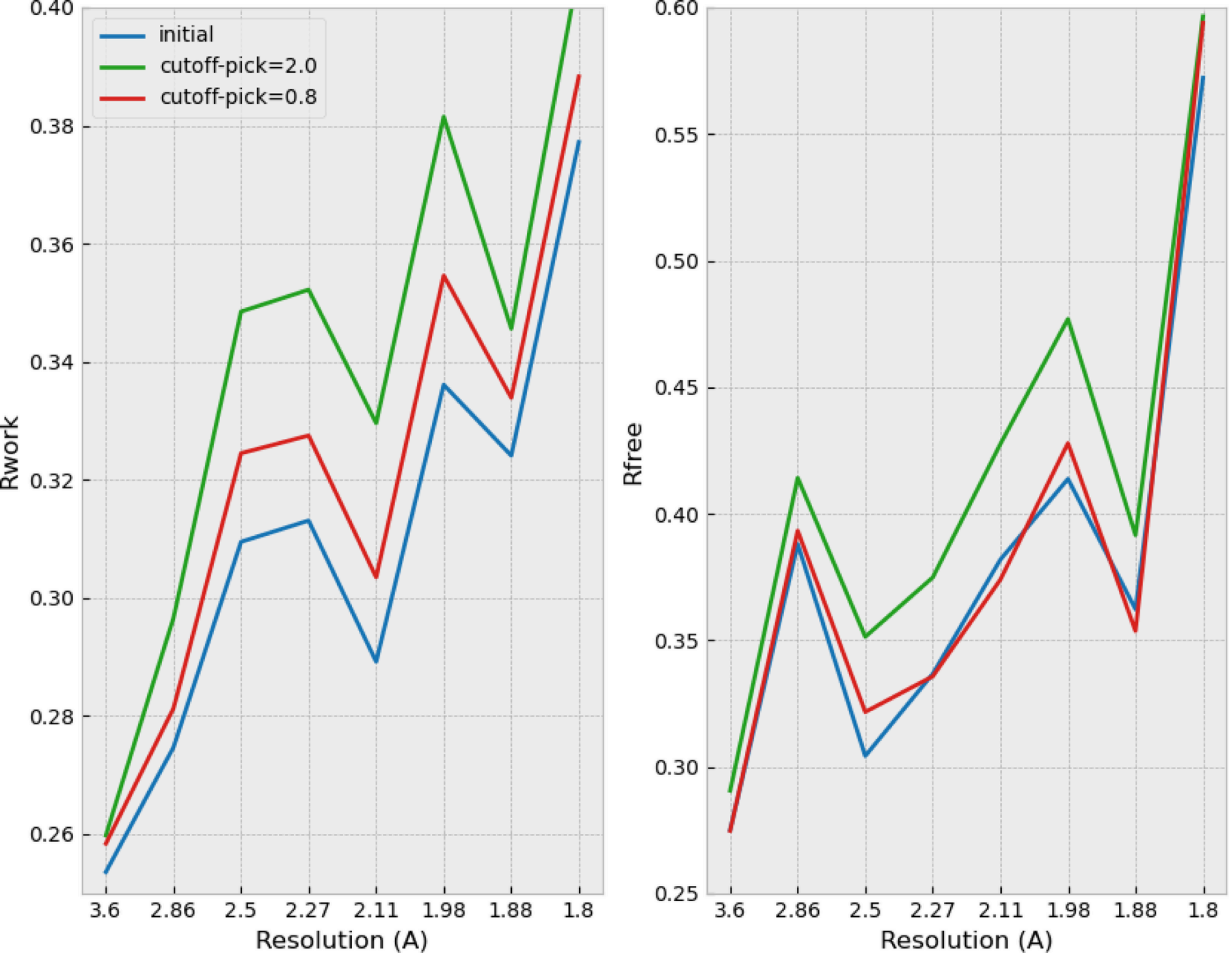
*R*_work_ and *R*_free_ crystallographic quality indicators at different resolution shells obtained from the complete dataset (11k frames) of HEWL + Ga. The quality of the sparsified data (at 0.8σ and 2.0σ) is compared with the initial dataset.

The same *R*_work_ and *R*_free_ quality indicators are reported in Fig. 5 ▸ as a function of the resolution shell in order to monitor if the degradation is uniform among shells or if it affects mostly the outer shells. These indicators compare the initial dataset with the sparsified ones at 0.8σ and 2.0σ, which were subsequently re-densified for the analysis.

Both *R*_work_ and *R*_free_ exhibit degradation as expected and this degradation is rather uniform over all shells; it does not affect the outer shells more. The degradation of *R*_free_ at moderate sparsification (0.8σ) is once again very limited.

#### 3.4.3. Ability to phase sparse data and quality of the density map

**Figure 6:**
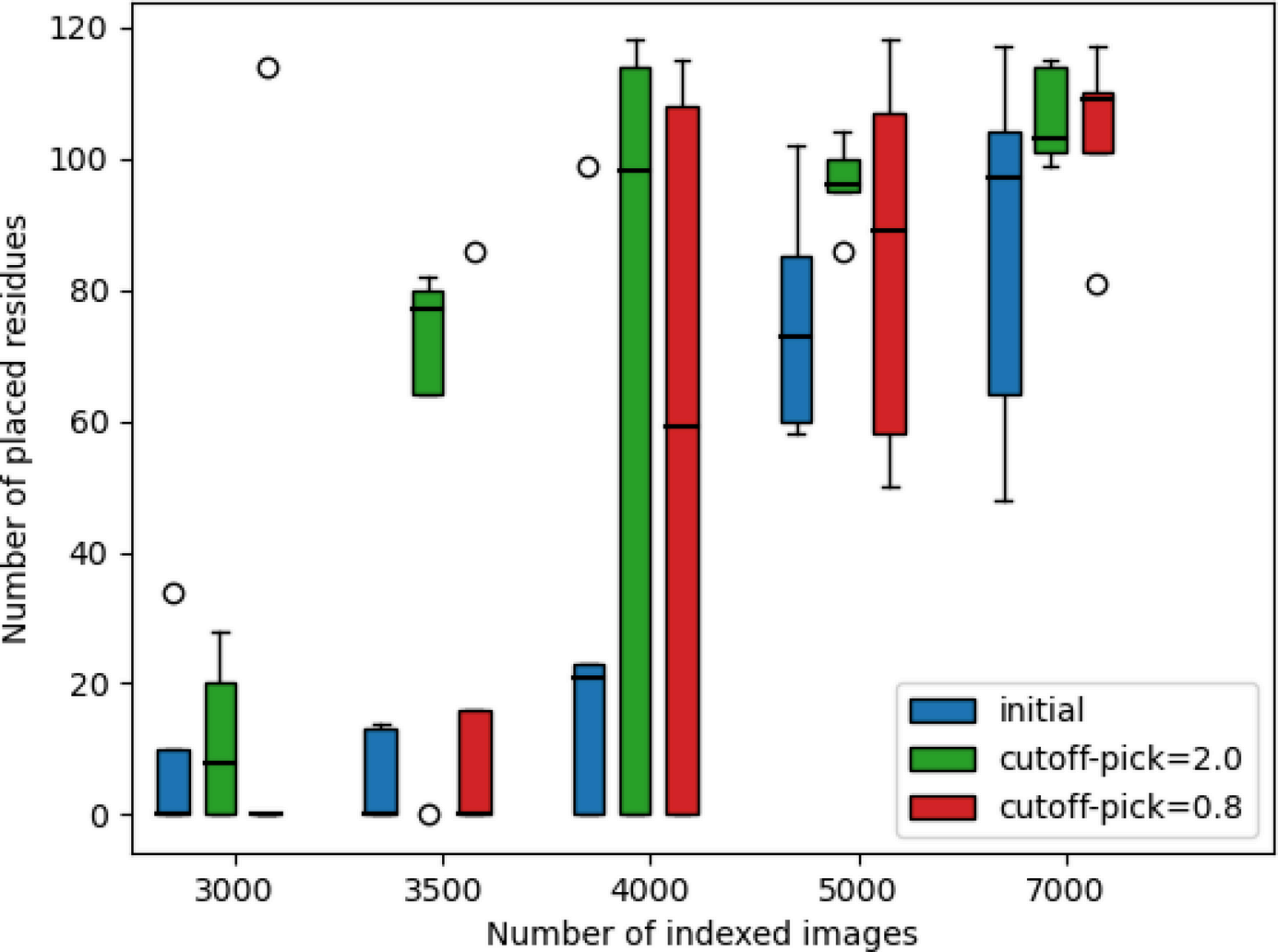
Influence of the sparsification (at 0.8σ and 2.0σ versus the initial dataset) on the ability to phase the HEWL + Ga protein with an artificially reduced number of frames, in order to limit the strength of the anomalous signal.

One of the main concerns with sparsification is that it may degrade the weak anomalous signal, which is precious for phasing. The HEWL + Ga dataset was truncated at different lengths (from 3000 to 7000 out of the 11 512 frames) in order to ‘artificially’ decrease the anomalous signal strength in the dataset. Fig. 6 ▸ shows the number of residues that were properly placed in the electron-density map with an automatic procedure.

The sparsified dataset does not show fewer residues properly placed after the procedure; it even looks marginally better than the initial dataset, especially for larger compression factors where the dataset could be phased with as few as 3500 frames. With Eiger detector data, a sparsification at 0.8σ offers 2× extra compression without noticeable degradation of the quality of the processed data.

### 3.5. Influence of the sparsification on the quality of serial crystallography data acquired with a Jungfrau detector

#### 3.5.1. Dataset description

**Figure 7:**
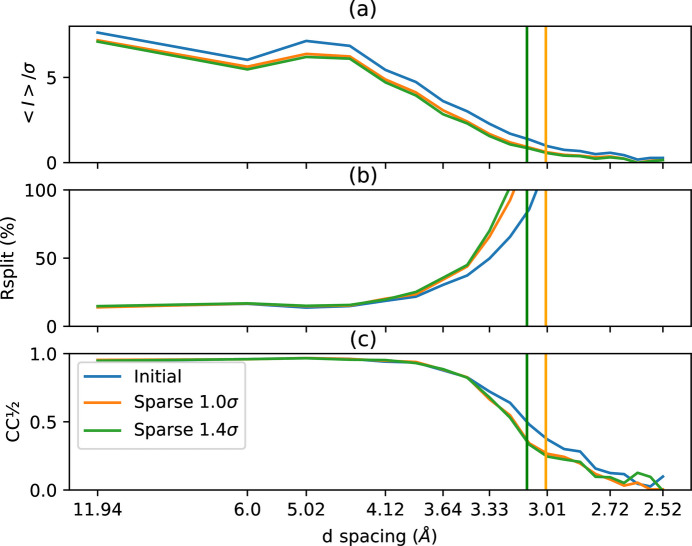
Comparison of crystallographic quality indicators as a function of the resolution shell for sparsified data (1.0σ in orange and 1.4σ in green) in comparison with the initial dense data (in blue): (*a*) SNR, (*b*) *R*_split_ and (*c*) CC_1/2_. The two vertical lines indicate the resolution shell at which the SNR drops below the sparsification threshold, *i.e.* the limit at which all signal is expected to be lost.

**Figure 8:**
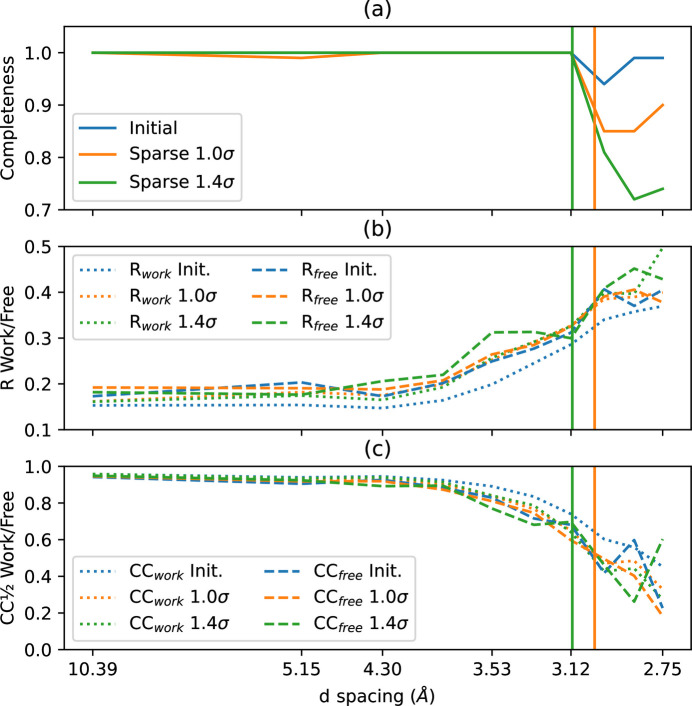
Comparison of crystallographic quality indicators in real space after refinement using *phenix.refine* as a function of the resolution shell for sparsified data (1.0σ in orange and 1.4σ in green) in comparison with the initial dense data (in blue): (*a*) completeness of the dataset, (*b*) *R*_work_ (dotted) and *R*_free_ (dashed), and (*c*) CC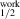 (dotted) and CC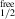 (dashed). The two vertical lines indicate the resolution shell at which the SNR drops below the sparsification threshold, *i.e.* the limit at which all signal is expected to be lost.

Small crystals of an NQO1 sample (Grieco *et al.*, 2024 ▸), complexed with NADH, were collected at ESRF-ID29 using a fixed-target sample environment and the Jungfrau 4M detector (PDB code 8rfm). The complete dataset represents 574k frames (5 TB), out of which 25 809 frames were selected with nice peaks (4.5% of the total). The indexing rate for dense data and two sparse datasets (cut-offs at 1.0σ and 1.4σ) is reported in Table 2 ▸, and the crystallographic quality indicators are reported in Fig. 7 ▸ in reciprocal space and Fig. 8 ▸ in real space.

#### 3.5.2. Data statistics

The processing was performed with *CrystFEL* (White *et al.*, 2012 ▸) (version 0.11) with the geometry of the detector optimized with the *Millepede* procedure (Blobel & Kleinwort, 2002 ▸; Kleinwort, 2021–2024 ▸). Indexing was performed with the default parameters of *xgandalf* (Gevorkov *et al.*, 2019 ▸) based on peak position from *peakfinder8* (Barty *et al.*, 2014 ▸).

From the 〈*I*〉/σ curve, Fig. 7 ▸(*a*), there is a systematically lower SNR from sparsified data in comparison with the initial dataset, regardless of the resolution shell. This could be due to too much noise being added when densifying the data, especially when this dataset was acquired (and processed) at the full energy resolution of the Jungfrau detector, here 485 ADUs per photon (in comparison, an Eiger detector has 1 ADU per photon). The SNR curve obtained on uncompressed data was used to assess the resolution shell at which there is no more signal expected to be saved, shown by the two vertical lines in green (1.4σ) and orange (1.0σ). On the right of these lines, there is supposed to be no more signal, while on the left, there is supposed to be no degradation of the signal in an ideal case. The evolution of *R*_split_ and CC_1/2_, plotted in Figs. 7 ▸(*b*) and 7 ▸(*c*), shows a degradation of the quality that is much earlier, by half an ångström, in comparison with what would have been expected.

#### 3.5.3. Refinement statistics

These data were finally refined in real space using *phenix.refine* (Afonine *et al.*, 2012 ▸) until a resolution of 2.7 Å, which is noticeably better than what can be expected from the sparse data (limited to 3.0 and 3.1 Å for 1.0σ and 1.4σ, respectively). The results are summarized in Fig. 8 ▸.

Fig. 8 ▸(*a*) shows the completeness of the dataset as a function of the resolution shell. It confirms that the sparsification algorithm works as expected, and that the signal starts to degrade where the SNR drops below the picking threshold. Fig. 8 ▸(*b*) superimposes *R*_work_ in dotted lines (full dataset) in comparison with *R*_free_ where the fit is performed on 95% of the dataset and the quality assessment is performed on the remaining 5% (dashed line). Thus, *R*_work_ is always expected to be better than *R*_free_. While *R*_work_ for the initial dataset looks much better than the sparsified version, the degradation is very limited for *R*_free_, especially between the initial and the sparsified dataset at 1σ. The same is observed for CC_1/2_ in Fig. 8 ▸(*c*): the initial dataset shows a clear degradation between the ‘work’ version and the ‘free’ version, but this degradation is less important for the sparsified version. The CC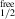 indicators hardly degrade between the initial dataset and the two sparsified ones, confirming the ability to solve the protein structure from sparse data.

### 3.6. Conclusions on sparsification

The sparsification algorithm presented here is generally applicable to any kind of single-crystal diffraction experiment where the background is isotropic. This excludes notably diffuse scattering experiments but is generally applicable to small-molecule crystallography and macromolecular crystallography, and even serial crystallography, when the images are nicely centrosymmetric and shadows are properly masked out. The additional compression offered by sparsification is especially interesting for serial crystallography, where datasets of millions of frames are collected per experiment. As for any lossy compression, the user still has the responsibility of choosing wisely the threshold level as it will limit later on the quality of the results one may extract from these data. This is of crucial importance for macromolecular crystallography where valuable information is still present in reflection with an SNR of less than one (Karplus & Diederichs, 2012 ▸). We have demonstrated that a sparsification at 0.8σ, which has an actual compression of 2.0× on Eiger data (2.6× for Jungfrau data), preserves nicely the electron-density map and is hardly distinguishable from the uncompressed data. Sparsification at 1.0σ, offering a 2.6× compression on Eiger data (3.2× for Jungfrau data), preserves enough signal to refine a protein with very limited degradation of the *R*_free_ and CC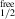 quality indicators. The Jungfrau data showed systematically larger compression rates for sparsification because data were collected with 485 ADUs per photon (and are tunable) while Eiger detectors always operate at 1 ADU per photon (*i.e.* they have less noise).

In contrast with *ROIBIN-SZ* (Underwood *et al.*, 2023 ▸), which preserves all pixels in the neighborhood of identified peaks and stores the background heavily binned, the sparsification presented here does not require a complete peak search; thus, it is simpler, with fewer parameters to be tuned, and is able to save any pixel that is intense enough. Nevertheless, peak finding is of crucial importance for identifying Bragg peaks, one of the first stages of any data-reduction pipeline.

## 4. Application to peak finding for serial crystallography

A classical way of pre-processing serial crystallography data is to shrink the amount of data by sieving out empty or bad frames, only keeping the frames that deserve processing. This is the role of the veto algorithm.

The sigma-clipping algorithm provides us with the background (average and deviation) and is used to pick pixels that are likely to be part of Bragg peaks, like *peakfinder8* (Barty *et al.*, 2014 ▸) does. For this, several additional checks are performed on a local neighborhood that is a small square patch (typically 3 × 3 or 5 × 5 pixels, user defined): (i) Is the considered pixel the maximum of the local neighborhood? (ii) Are enough pixels of the local neighborhood satisfying the SNR condition (user-defined parameter)?

**Figure 9:**
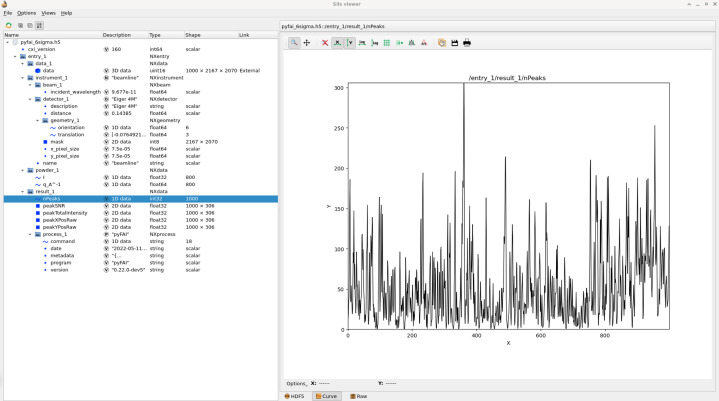
A peak-picking CXI file produced by *pyFAI* and visualized with the *silx viewer* (Vincent *et al.*, 2021 ▸). The left-hand side contains the HDF5 tree structure while the right-hand side presents the default plot with the number of peaks found per frame.

For each peak, the coordinates of the centroid, the sum of data and its propagated deviation are recorded and reported. These peak positions are saved into an HDF5 file (as represented by Fig. 9 ▸) following the CXI format (Maia, 2012 ▸), which can be read from *CrystFEL* (White *et al.*, 2012 ▸). *CrystFEL* allows one to swap peak-picking algorithms (Zaefferer, 2000 ▸; Barty *et al.*, 2014 ▸; Hadian-Jazi *et al.*, 2021 ▸) and indexing tools (Kabsch, 2010 ▸; Powell *et al.*, 2013 ▸; Ginn *et al.*, 2016 ▸; Gevorkov *et al.*, 2019 ▸, 2020 ▸).

The serial crystallography beamline at ESRF (ID29) uses a Lima2 monitor (Debionne *et al.*, 2022*a* ▸) as a visualization tool. It is inspired by *NanoPeakCell* (Coquelle *et al.*, 2015 ▸) for online visualization and feeds back information to check if peaks found actually correspond to the crystal lattice expected for the sample. We will first compare the peak-picking algorithm with some reference implementation on a single frame before evaluating the quality of the picked points on a serial crystallography dataset.

### 4.1. Comparison of picked peaks

**Figure 10:**
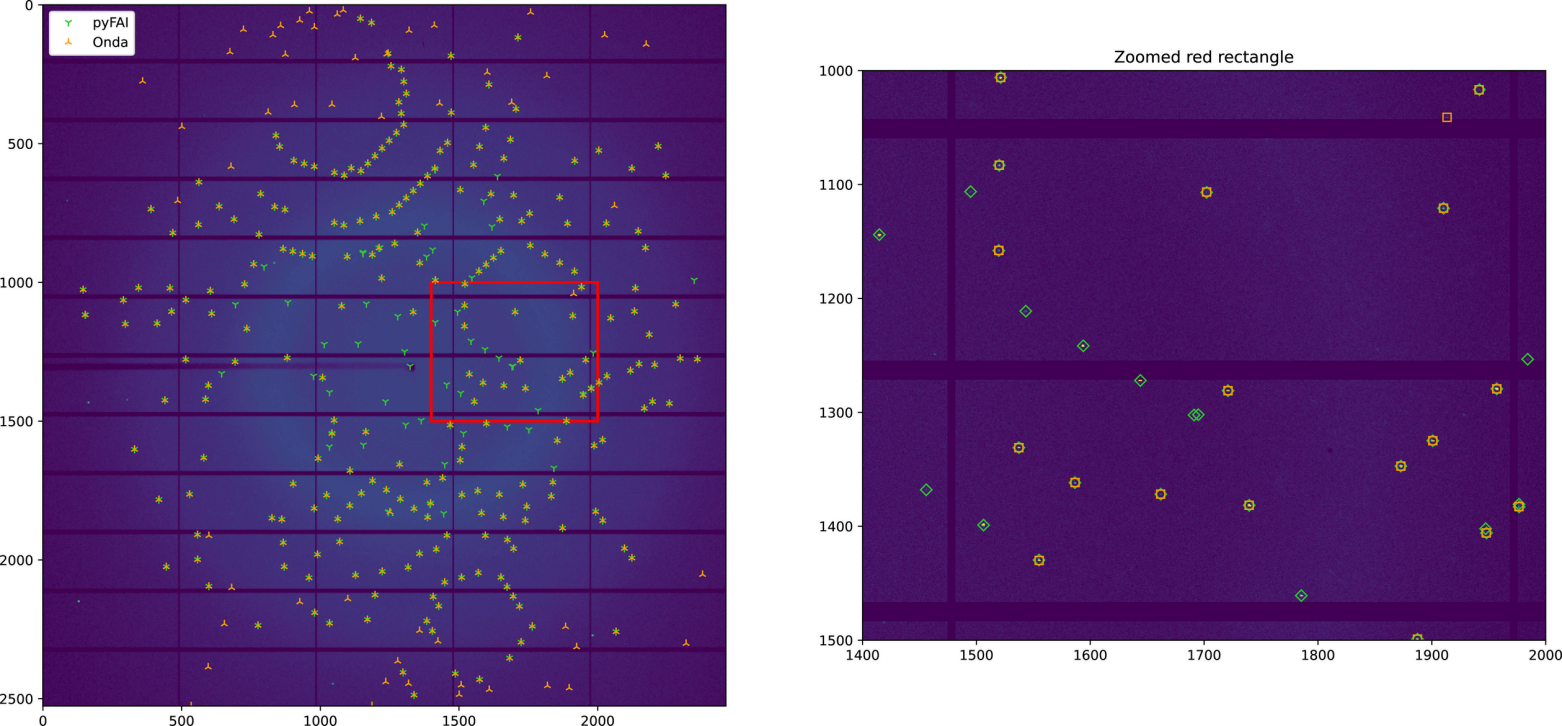
Comparison of the reference *peakfinder8* interfaced with *OnDA* (in orange, execution time of 300 ms) and the version from *pyFAI* (in green, execution time of 10 ms) on top of a Pilatus 6M diffraction frame of an insulin crystal. The subplot on the right is a close-up of the red rectangle.

Fig. 10 ▸ presents a comparison between the original *peakfinder8* described by Barty *et al.* (2014 ▸), interfaced in Python via *OnDA* (Mariani *et al.*, 2016 ▸), and the version implemented in *pyFAI* on the same Pilatus 6M image already used in Fig. 2 ▸.

In Fig. 10 ▸, most peaks found by both implementations match and correspond to Bragg reflections; the close-up on the right allows one to visualize Bragg spots in the image. There are more green peaks (found by *pyFAI*) closer to the beam center, while more orange peaks (found by *OnDA*) are located in the outer shell. This plot was made with a minimum SNR of 3 and a noise level of 1, since the Pilatus detector is Poissonian.

**Figure 11:**
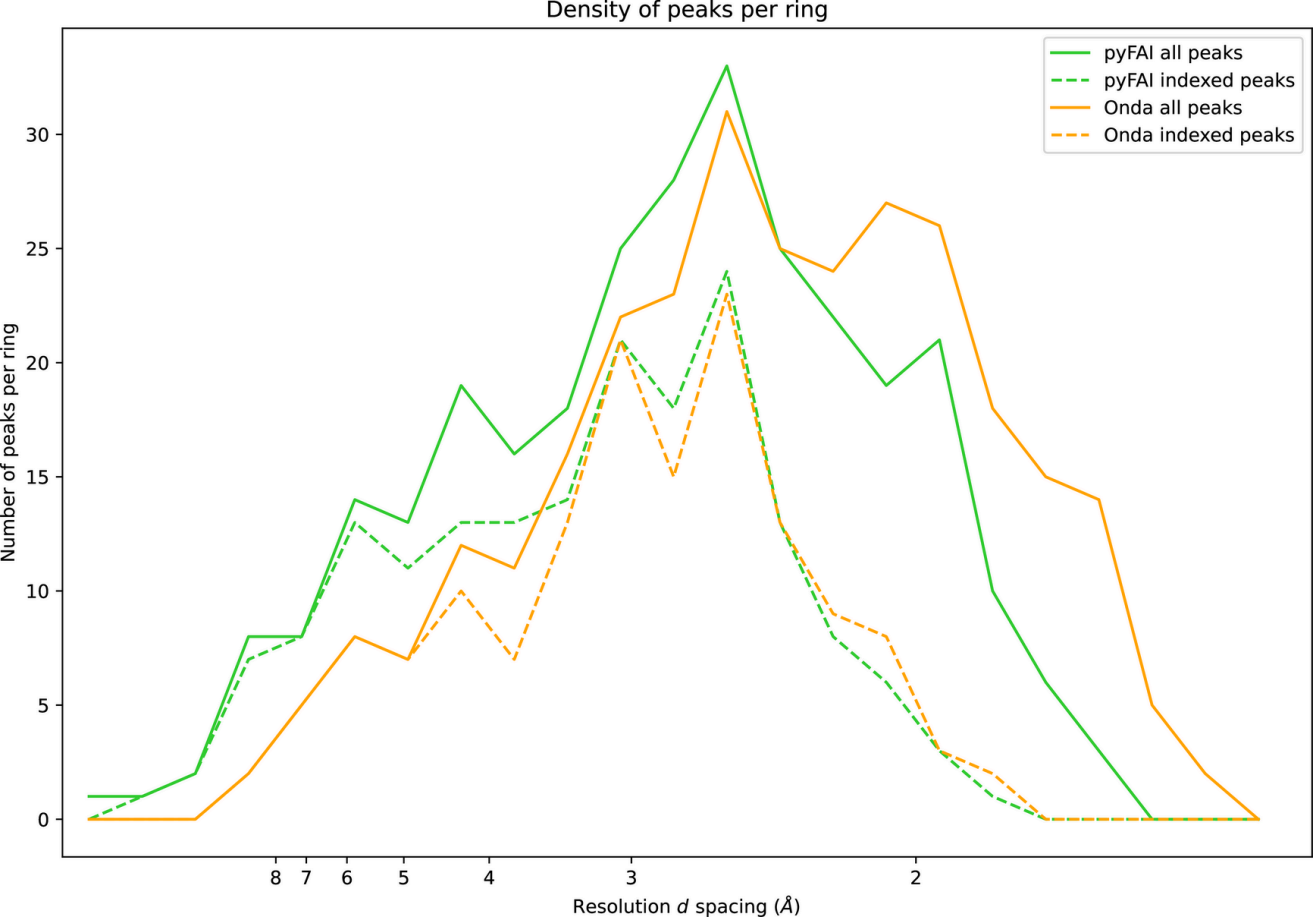
Number of peaks found in the different resolution shells for the peak finders implemented in *pyFAI* and in *OnDA*. The width of each radial shell is 2 nm^−1^ in *q* space. The dashed lines represent the number of these peaks that have successfully been indexed, *i.e.* located at less than two pixels away from an expected reflection (calculated by *xgandalf*).

Peaks were registered if four pixels met the SNR criterion in a 5 × 5 pixel patch around the peak. These parameters were tuned to obtain a comparable number of peaks with both implementations: 290 with *pyFAI* and 293 with *OnDA*. The similarity of these figures enables a direct comparison of peaks found per resolution shell, histograms that are plotted in Fig. 11 ▸.

Fig. 11 ▸ presents histograms of *q* values (modulus of the scattering vector) of the peaks found with different methods, *i.e.* the number of peaks having a given *q* value. For readability, these histograms (bin width of 2 nm^−1^) have been represented as plots with the horizontal axis labeled in *d* spacing (*q* = 2π/*d*). The analysis of these histograms confirms that the implementation in *pyFAI* is getting more points closer to the beam center while the reference implementation is picking more points at larger *q* values. This is probably due to the curvature of the Debye–Scherrer ring: the original version of *peakfinder8* evaluates the variance in a neighborhood defined by some radius around the point of interest and the variance is higher close to the beam center because of the substantial curvature of these rings. On the other hand, *pyFAI* knows about this curvature and measures the variance along the ring. Points picked by *OnDA* at larger *q* values do not look like Bragg peaks, but this could be a side effect of the parameter tuning to get the same number of peaks for both algorithms.

The same figure presents, with dashed lines, the number of peaks that are coincident (within two pixels) with expected reflections (after indexing using *xgandalf*). This demonstrates that the additional peaks found by *pyFAI* in the inner shell (*d* > 3Å) are consistent with Bragg peaks and thus are valuable for indexing, while the additional peaks found by *OnDA* in the outer shell (*d* < 2Å) are not Bragg peaks and thus are detrimental.

A word on performance: the Python binding in *OnDA* to the *peakfinder8* algorithm from *Cheetah* runs in 180 ms on a high-end server CPU (AMD Epyc 7262) and 300 ms on a workstation (Intel Xeon E5-1650 v4). The version available in *pyFAI* was designed in OpenCL (Khronos, 2008 ▸; Stone *et al.*, 2010 ▸; Klöckner *et al.*, 2012 ▸) and runs best on GPUs: 30 ms on an AMD Vega 56 and 10 ms on a Nvidia RTX A5000.

### 4.2. Quality of the *peakfinder* algorithm on serial crystallography data

The quality of peaks extracted with this algorithm was evaluated on a serial crystallography dataset. A subset of 1000 frames of the dataset used in Section 3.4 was used as a probe and was indexed with the *indexamajig* tool from *CrystFEL*. Since all frames show Bragg peaks (Fig. 9 ▸), the number of indexed frames can be seen as a quality indicator of the peak-picking algorithm used, when all other parameters remain unchanged. The indexing was performed with the *xgandalf* algorithm (Gevorkov *et al.*, 2019 ▸) with default settings from *CrystFEL* v0.10.1 and was provided with the following cell parameters: tetragonal 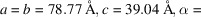

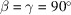
. Table 3 ▸ compares the number of frames properly indexed with the different picking algorithms available in *CrystFEL* and with the algorithm presented here.

Since the Eiger detector is a counting detector, the global threshold for algorithms *zaef* and *peakfinder8* had to be lowered (to 50, which is the maximum of the background signal on any frame), and the default SNR value was used for these algorithms. The same SNR value of 5 was used for *pyFAI*. Default parameters were used for the *peakfinder9* and *RobustPeakFinder* algorithms. The reported runtime corresponds to the execution time on a single core of an Intel Xeon Gold 6134.

The indexing rate obtained with the algorithm from *pyFAI* is on par with the reference implementations like *peakfinder8* or *peakfinder9* available from *CrystFEL*. Since time is mostly spent in indexing, sets of peaks that are simpler to index present a lower runtime as fewer retries are needed. Retries can be deactivated but they increase significantly the success of indexing. Table 3 ▸ also presents the indexing performances when using the option *–xgandalf-fast-execution* from *indexamajig*, which is five to six times faster and exhibits a limited degradation of the indexing rate. The peak extraction in *pyFAI* is about as fast as the sparsification, so it can be used online to perform the pre-analysis and provide peaks to *NanoPeakCell*. Nevertheless, the executable peak finder available from *pyFAI* (offline tool) has a total execution time that is much larger: about 30 s for 1000 frames, most of which is spent in reading and writing the different HDF5 files.

### 4.3. Peak count as a veto algorithm

Since the background extraction and peak finding are performed in real time on the serial crystallography beamline ID29 at ESRF, the information about the number of Bragg spots can be used to assess the quality of each individual image and the acquisition system can decide to discard a frame depending on the number of peaks and a live-adjustable threshold.

**Figure 12:**
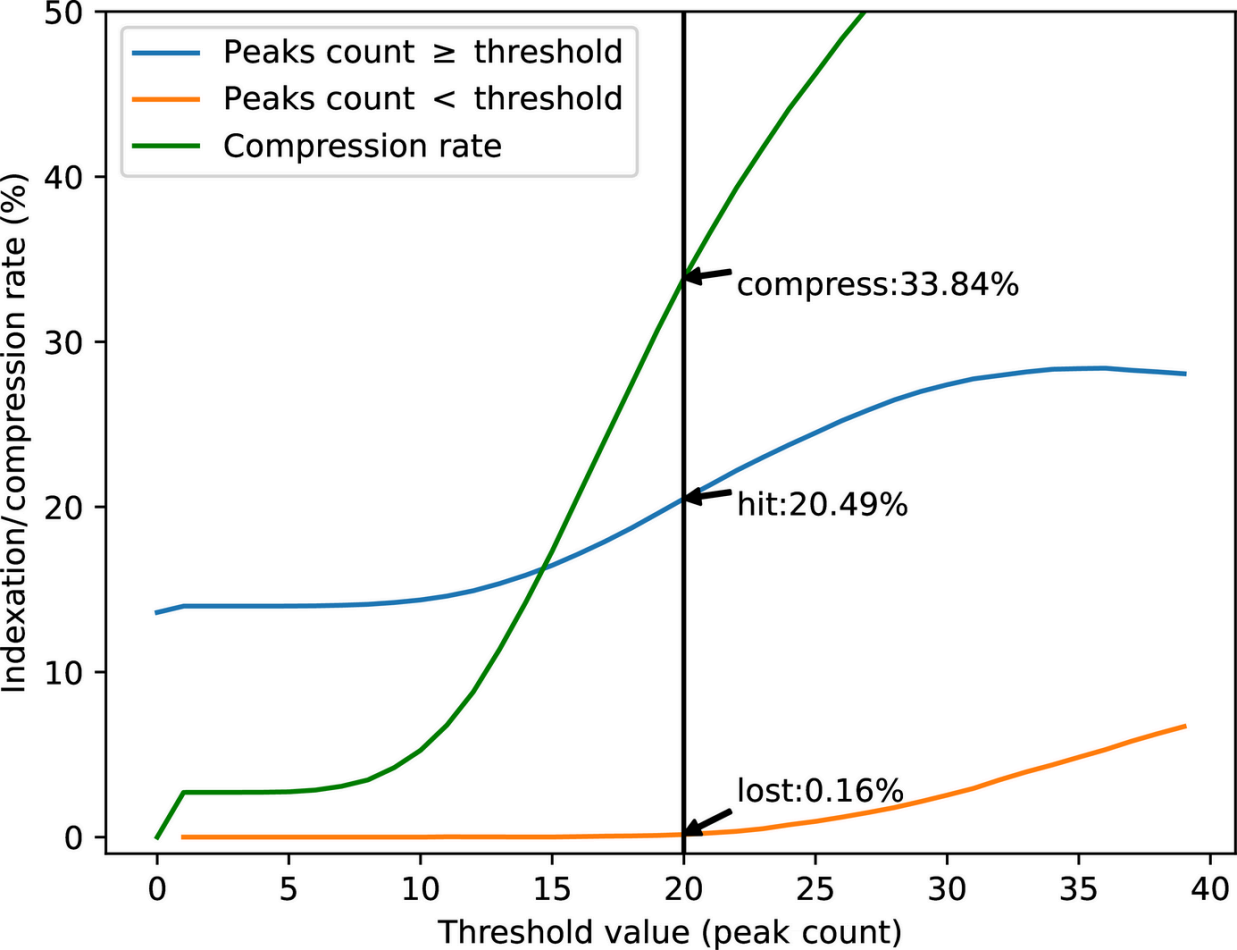
Indexing rates of frames that would be considered as ‘hit’ or ‘non-hit’, as a function of the peak-count threshold. The green curve represents the disk space that could have been saved. The sample dataset consists of 80 000 frames of lysozyme micro-crystals indexed off-line with *xgandalf* in *CrystFEL*.

Since the beginning of operation of ESRF-ID29 in 2022, the beamline has operated with the veto algorithm deactivated; the number of peaks found was just recorded for future exploration. Here, Fig. 12 ▸ presents the indexing rate of ‘hit’ (in blue) and ‘non-hit’ frames (in orange) when changing the threshold for the minimum peak-count per frame. The expected compression rate is displayed in green. The dataset consists of 80 000 lysozyme micro-crystals between two mylar films, raster scanned with an X-ray beam of 11.56 keV at ESRF-ID29. The offline analysis was performed with *CrystFEL* v0.11.0 using *peakfinder8* and *xgandalf* as indexers.

Since the Jungfrau detector is an integrating detector, it has relatively more background noise than a photon-counting detector: here, data were saved with 8 ADUs per photon, making the compression rates of hit and non-hit frames similar. In this example, discarding frames with fewer than 20 peaks would have allowed one to save one-third of the network bandwidth and disk space, losing only 0.16% of indexable frames. The indexing rate of actually recorded frames also increased to 20%. It is noticeable that the hit rate was especially high in this experiment, with 84% of frames showing diffraction peaks. One would not expect a user experiment to have such a high concentration of crystals, and in normal conditions the expected compression ratio should be higher. The veto algorithm, having proved its robustness, is now activated for most experiments, thanks to graphical helper tools, to assess the optimal threshold during the experiment and allow one to set these parameters on the fly for real-time processing.

### 4.4. Limitations

There is a strong sensitivity in the indexing rate with data from the Jungfrau detector, related to the description of the detector in *CrystFEL* and in *pyFAI*. The Jungfrau 4M detector is built from eight modules manually assembled and exhibits some residual misalignment, of the order of a few pixels, and mis-behaving pixels. This misalignment is much larger than what is commonly encountered with Eiger detectors from Dectris, where misalignment is usually less than one pixel in size (75 µm). For Eiger detector data, where the detector is defined as a single rigid module, the indexing rate is fairly independent of the peak-picking algorithm as described in Section 4.2. This means that the peak positions provided by the veto algorithm are suitable for indexing and this saves reading of the complete frame if it cannot be indexed.

With data from the Jungfrau detector, the indexing rate was lower than with certain peak-finder algorithms integrated into *CrystFEL*. Work is ongoing to convert the geometry description from *pyFAI*, where every pixel is independent, to the geometry used in *CrystFEL*, and *vice versa*. We found this difference of indexing was related to the description of the mask and of pixel position in different software. Until this issue is addressed, the safest solution is to re-extract peak positions for indexing using one of *CrystFEL*’s provided peak-finding algorithms.

## 5. Conclusions

Background analysis of single-crystal diffraction images can be implemented efficiently using iterative azimuthal integration, which allows the separation of the signal originating from Bragg peaks from isotropic background.

A lossy compression algorithm for diffraction frames, called sparsification, has been built on top of this signal separation, with the average background saved on one side and the position and intensity of the most intense pixels (probably belonging to peaks) on the the other. The quality of the compression has been demonstrated on macromolecular rotational and serial crystallography data. The degradation of the signal has been monitored after a compression–decompression cycle, both in reciprocal space using crystallographic quality indicators and after Fourier transform in direct space, where the number of residues placed automatically was checked. The degradation of the signal was also monitored as a function of the threshold, and sparsification at 0.8σ–1.0σ still enables one to reconstruct the molecular structure of proteins, with both Eiger and Jungfrau detectors.

The second application presented here is a peak finder for serial crystallography that locates peak positions in real time and can be used as a veto algorithm to discard images without (enough) diffraction peaks. The peaks picked were evaluated against state-of-the-art peak-finder algorithms like *peakfinder8* and the results were comparable in quality, while much faster thanks to the usage of a GPU. This veto algorithm is now used in production at the ESRF serial crystallography beamline (ID29) to save storage space.

One of the strengths of this peak-finder algorithm is that it is optimized to work on GPUs. It is thus ideally suited to be coupled with the next generation of crystal indexer (Gasparotto *et al.*, 2024 ▸), which is running on the same kind of hardware, allowing one to save two memory transfers per frame and maybe achieve real-time integration of serial crystallography data.

## Figures and Tables

**Table 1:** Quality indicators after peak integration and averaging using *XDS* (Kabsch, 2010 ▸) The lysozyme (HEWL) dataset was provided by Dectris for advertising their Eiger 4M detector (Dectris, 2014 ▸).

	Initial dataset			Lossy compressed dataset (2σ)		
Indicator	2.91 Å	2.06 Å	All	2.91 Å	2.06 Å	All
Completeness	98.8	90.8	93.8%	99.8	90.6	93.5%
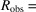 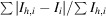	9.9	57.3	12.5%	9.2	61.2	11.4%
*R*_expected_	8.8	73.2	15.0%	8.2	68.7	12.1%
*R*_meas_ †	10.3	61.2	13.2%	9.6	65.5	12.0%
CC_1/2_‡	99.7	94.0	99.7	99.6	95.4	99.7
〈*I*/σ〉	25.80	5.39	10.52	26.33	4.09	10.17

† Diederichs & Karplus (1997 ▸).

‡ Karplus & Diederichs (2012 ▸).

**Table 2:** Indexing statistics of the NQO1 dataset (25 809 frames, 4.5% of the complete dataset)

	Dense	Sparse 1.0σ	Sparse 1.4σ
Disk space (MB)	166	52	32
Compression	1×	3.2×	5.2×
Frames indexed	22874	20176	20384
Indexing rate (%)	88.6	78.2	79.0

**Table 3:** Indexing rate obtained with *xgandalf* (Gevorkov *et al.*, 2019 ▸) from peak positions extracted with different picking algorithms available from *CrystFEL* (White *et al.*, 2012 ▸) on a subset of 1000 frames of microcrystals of lysozyme (HEWL + Ga) collected with an Eiger 4M detector at ESRF-ID30A3

	*xgandalf* (default)		*xgandalf* (fast)	
Peak-picking method	Index rate (%)	Runtime (s)	Index rate (%)	Runtime (s)
*zaef* †	10.0	2178	10.0	430
*peakfinder8* ‡	49.5	10397	48.5	1757
*peakfinder9* §	44.2	8328	43.5	1436
*RobustPF* ¶	22.4	6314	21.2	1628
*pyFAI* ††	49.7	9325	49.2	1595

† Zaefferer (2000 ▸).

‡ Barty *et al.* (2014 ▸).

§ Gevorkov *et al.* (2024 ▸).

¶ Hadian-Jazi *et al.* (2021 ▸).

†† This contribution.

## Footnotes

1 See https://github.com/silx-kit/pyFAI/blob/main/doc/source/usage/tutorial/Variance/uncertainties.ipynb.
